# Exploiting Oxidative Stress as Achilles’ Heel: From Redox Homeostasis to Ferroptosis in Prostate Cancer

**DOI:** 10.3390/antiox14121517

**Published:** 2025-12-18

**Authors:** Sanghyeon Yu, Jihyun Baek, Taesoo Choi, Man S. Kim

**Affiliations:** 1 Translational-Transdisciplinary Research Center, Clinical Research Institute, Kyung Hee University Hospital at Gangdong, Kyung Hee University College of Medicine, Seoul 05278, Republic of Korea; sanghyeon99@khu.ac.kr; 2 Department of Biomedical Science and Technology, Graduate School, Kyung Hee University, Seoul 02453, Republic of Korea; 3 Division of Nephrology, Department of Internal Medicine, CHA Bundang Medical Center, CHA University, Seongnam 13496, Republic of Korea; spreesh7@chamc.co.kr; 4 Department of Urology, Kyung Hee University Hospital at Gangdong, College of Medicine, Kyung Hee University, Seoul 05278, Republic of Korea; howdyhowdy@khu.ac.kr; 5 Center for Space Biomedical Science, NEXUS Institute, Kyung Hee University, Yongin-si 17104, Republic of Korea

**Keywords:** prostate cancer, ferroptosis, oxidative stress, androgen receptor, GPX4, FSP1, MBOAT2, SLC7A11, castration resistance, precision oncology

## Abstract

Prostate cancer remains a leading cause of cancer-related mortality and castration-resistant prostate cancer (CRPC) is a critical therapeutic challenge. This review establishes a conceptual framework analyzing ferroptosis vulnerability through two principles: “robustness through redundancy” in defense systems and the “evolutionary arms race” between androgen receptor (AR) signaling and oxidative resistance. We traced the evolutionary trajectory of hormone-sensitive diseases, where the AR coordinates ferroptosis defenses via *SLC7A11*, *MBOAT2*, and *PEX10* regulation through progressive adaptations: AR-V7 splice variants that maintain defense independently of androgens, AR amplification conferring hypersensitivity, and AR-independent JMJD6-ATF4 bypass in SPOP-mutated tumors. This transforms ferroptosis from a static vulnerability to a stage-specific strategy. Novel approaches include menadione-based *VPS34* targeting, which induces triaptosis through an oxidative endosomal catastrophe. We categorized the rational combinations mechanistically as vertical inhibition (multi-step targeting of single pathways), horizontal inhibition (synthetic lethality across parallel defenses), and vulnerability induction (creating exploitable dependencies). Ferroptosis-induced immunogenic cell death enables synergy with checkpoint inhibitors, potentially transforming immunologically “cold” prostate tumors. This review establishes ferroptosis targeting as a precision medicine paradigm exploiting the tension between the oxidative requirements of cancer cells and their evolved, yet architecturally vulnerable, defense systems, providing a framework for stage-specific, biomarker-guided interventions.

## 1. Introduction

### 1.1. Prostate Cancer (PCa): Epidemiology and Therapeutic Challenges

PCa is the leading cause of cancer-related mortality in men worldwide. According to the Global Burden of Disease Study 2023, PCa continues to cause substantial morbidity across diverse geographic regions [1]. Current standard-of-care treatments rely primarily on androgen deprivation therapy (ADT), which targets the androgen receptor (AR) signaling axis. However, virtually all patients develop resistance within 18–24 months, which progresses to castration-resistant PCa (CRPC) [2]. Despite the use of novel AR-targeting agents (enzalutamide, apalutamide, and darolutamide) and taxane chemotherapies, the five-year survival rate for metastatic prostate cancer remains approximately 30% [3].

Therapeutic resistance arises from multiple interconnected factors: AR splice variants (particularly AR-V7) enable ligand-independent AR activity [4], metabolic reprogramming allows adaptation to therapeutic pressure [5], and the immunosuppressive tumor microenvironment facilitates treatment evasion [6]. These unmet clinical needs necessitate the development of novel therapeutic strategies that exploit previously untargeted vulnerabilities.

### 1.2. Redox Homeostasis: A Double-Edged Sword

Cancer cells maintain elevated basal reactive oxygen species (ROS) levels compared to normal cells due to mitochondrial dysfunction, increased metabolic activity, and oncogenic signaling [7]. This creates a fundamental “oxidative paradox”: at physiological concentrations, ROS function as essential signaling molecules regulating proliferation, survival, and metastasis [8]. However, excessive ROS accumulation triggers oxidative damage to proteins, lipids, and DNA, resulting in cell death [9].

Cancer cells navigate this balance through adaptive upregulation of antioxidant defenses, particularly the glutathione (GSH) system and *NRF2* [10]. This elevated antioxidant capacity confers resistance to both endogenous oxidative stress and pro-oxidant therapies. Thus, the oxidative paradox presents both opportunities and challenges: cancer cells require elevated ROS for proliferation yet remain vulnerable to strategies that increase oxidative stress beyond tolerable thresholds or impair compensatory defenses.

### 1.3. Ferroptosis: Oxidative Stress-Driven Cell Death

Ferroptosis is a distinctive form of regulated cell death characterized by iron-dependent accumulation of lipid peroxides [11]. Unlike apoptosis, ferroptosis features mitochondrial shrinkage, increased membrane density, and the absence of caspase activation [12]. Cellular labile iron pools catalyze oxidation of polyunsaturated fatty acid (PUFA)-containing phospholipids, particularly those containing arachidonic or adrenic acid [13,14]. Ferroptosis is biochemically distinct from other cell death pathways including apoptosis and necroptosis through its unique dependence on iron-catalyzed lipid peroxidation (Table 1).

Glutathione peroxidase 4 (GPX4) functions as a master ferroptosis regulator by reducing phospholipid hydroperoxides using GSH as a cofactor [15]. *GPX4* inactivation sensitizes cells to ferroptosis. Recent discoveries revealed parallel defense systems: ferroptosis suppressor protein 1 (FSP1) reduces coenzyme Q10 to trap lipid peroxyl radicals [16], while aldehyde dehydrogenase 7A1 (ALDH7A1) generates membrane NADH supporting *FSP1* activity and detoxifies reactive aldehydes [17].

In PCa, ferroptosis has therapeutic potential: PCa cells depend on lipid metabolism, AR signaling regulates ferroptosis defense mechanisms (*SLC7A11* and *MBOAT2*) [18], and novel ferroptosis inducers demonstrate marked preclinical efficacy [18,19]. Moreover, ferroptosis can circumvent apoptotic resistance and trigger immunogenic cell death, potentially synergizing with immunotherapy [20].

## 2. Cellular Sources and Regulation of Oxidative Stress

### 2.1. ROS Generation in Cancer Cells

Mitochondria represent the primary ROS source in cancer cells, generating superoxide radicals at Complexes I and III [21]. Cancer cells amplify ROS production through oncogene-driven metabolic reprogramming (RAS and MYC) and mitochondrial DNA mutations [22,23]. NADPH oxidases (NOX family), particularly *NOX4*, constitute secondary ROS sources in cancer cells, and NOX expression is frequently elevated in PCa [24]. Metabolic reprogramming further influences ROS homeostasis: the pentose phosphate pathway generates NADPH for antioxidant defense [25], while glutamine metabolism provides glutamate for GSH synthesis [26].

### 2.2. Antioxidant Defense Systems

#### 2.2.1. The GSH System

The GSH system is the primary oxidative defense mechanism. GSH synthesis proceeds through glutamate–cysteine ligase (GCL) and GSH synthetase, with cysteine availability governed by system Xc^−^ (SLC7A11) [27]. In PCa, AR transcriptionally regulates SLC7A11, linking hormonal status to susceptibility to ferroptosis [19]. *GPX4* occupies a unique position as the sole enzyme that reduces complex phospholipid hydroperoxides within the membrane. Its expression predicts ferroptosis sensitivity, with high *GPX4* conferring resistance [28].

Beyond AR-mediated transcriptional control, *SLC7A11* is regulated through multiple additional mechanisms in PCa. At the transcriptional level, the homeobox transcription factor (HOXA13), frequently overexpressed in metastatic PCa, directly binds to both *SLC7A11* and *SLC3A2* promoters to enhance System Xc^−^ activity [29]. Post-transcriptionally, *SLC7A11* expression is modulated through alternative splicing; the circular RNA circCNOT6L acts as a miR-143-5p sponge to upregulate the splicing factor (SRSF2), which promotes mature *SLC7A11* mRNA generation [30]. Similarly, *circATP2C1* sponges miR-654-3p to relieve translational repression of *SLC7A11*, activating the *SLC7A11/GPX4* axis [31]. At the post-translational level, the chimeric circular RNA *CCDC7_19–13_* encodes a novel protein *CCDC7_241__aa_* that promotes *TRIM21*-mediated K48-linked ubiquitination and proteasomal degradation of *SLC7A11* [32]. These multi-layered regulatory mechanisms highlight *SLC7A11* as a convergent node for ferroptosis control in PCa.

#### 2.2.2. FSP1-CoQ10 Axis

*FSP1* (AIFM2) operates as a *GPX4*-independent ferroptosis suppressor by reducing coenzyme Q10 (CoQ10) to ubiquinol using NADH, thereby trapping lipid peroxyl radicals [16]. Unlike the *GPX4*-GSH system that directly reduces phospholipid hydroperoxides, *FSP1* functions at the plasma membrane where it prevents lipid peroxidation through a distinct radical-trapping mechanism. *FSP1* undergoes liquid–liquid phase separation to form membrane condensates that concentrate *CoQ10* and enhance reduction efficiency [16]. Disruption of *FSP1* phase separation abolishes ferroptosis protection, establishing this structural organization as a potential therapeutic target. The existence of parallel defense systems—*GPX4*-GSH, *FSP1-CoQ10*, *ALDH7A1*, and *MBOAT1/2*—exemplifies biological redundancy, suggesting that combined inhibition of multiple pathways may achieve synthetic lethality where single-pathway targeting fails.

#### 2.2.3. ALDH7A1-Mediated Protection

**Figure 1:**
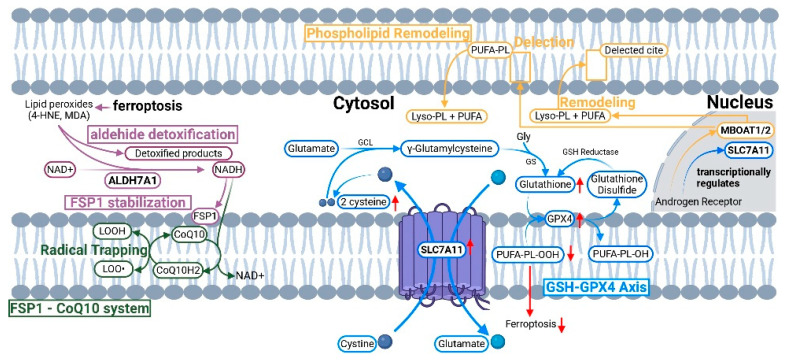
Molecular mechanisms of ferroptosis defense pathways in prostate cancer cells. The diagram illustrates three interconnected defense systems: the GSH-*GPX4* axis (blue pathway, center), *FSP1*-*CoQ10* system (green pathway, left), and phospholipid remodeling pathway (orange pathway, top right). The androgen receptor (AR) transcriptionally regulates *SLC7A11* and *MBOAT1/2* (shown in nucleus, right). Red arrows indicate pro-ferroptotic events (lipid peroxidation, PUFA-PL accumulation). Blue arrows indicate anti-ferroptotic protection (GSH synthesis, lipid peroxide reduction, radical trapping). *ALDH7A11* supports *FSP1* through membrane NADH generation and detoxifies reactive aldehydes (4-HNE, MDA). This integrated system exemplifies “robustness through redundancy” requiring multi-pathway targeting for effective ferroptosis induction.

*ALDH7A1* (aldehyde dehydrogenase 7A1) represents a recently discovered ferroptosis defense mechanism operating through membrane NADH generation and *FSP1* stabilization (Figure 1) [28]. *ALDH7A1* localizes to cellular membranes where it generates NADH from NAD^+^, supporting *FSP1*-mediated *CoQ10* reduction and lipid peroxyl radical trapping. This NADH supply mechanism enhances *FSP1* stability through phase separation, creating localized antioxidant microdomains.

The *AMPK-ALDH7A1-FSP1* axis links metabolic stress sensing to ferroptosis defense [28]. Additionally, *ALDH7A1* detoxifies reactive aldehydes (4-HNE, MDA) generated during lipid peroxidation, preventing protein modification. This dual function—supporting *FSP1*-CoQ10 activity while eliminating toxic byproducts—positions *ALDH7A1* as a critical node integrating metabolic adaptation with ferroptosis resistance.

#### 2.2.4. NRF2 and Additional Defenses

*NRF2* coordinates transcriptional responses to oxidative stress by inducing GSH synthesis enzymes, *SLC7A11*, and detoxification proteins [10]. Cancer cells frequently exhibit constitutive *NRF2* activation, contributing to therapy resistance. Additional ferroptosis suppressors include the thioredoxin system [33] and 7-dehydrocholesterol, which traps lipid peroxyl radicals through its conjugated diene structure [34].

### 2.3. Oxidative Damage: Molecular Consequences

Oxidative stress induces damage across multiple molecular targets. Cysteine residues undergo progressive oxidation to sulfenic, sulfinic, and sulfonic acids. Menadione preferentially oxidizes translation machinery components (eukaryotic initiation and elongation factors), disrupting protein synthesis and amplifying cellular dysfunction [35]. PUFA-containing phospholipids are particularly vulnerable due to bis-allylic methylene groups with low C-H bond dissociation energy [36]. In ferroptotic cells, high PUFA-PL content (from *ACSL4/LPCAT3* activity), elevated iron, and impaired *GPX4* function create optimal conditions for lipid peroxidation propagation [37], ultimately causing membrane rupture [12]. Oxidative DNA lesions, particularly 8-oxo-deoxyguanosine (8-oxo-dG), accumulate in PCa and correlate with disease stage [38,39]. Chronic oxidative stress overwhelms repair capacity, driving mutation accumulation and resistance development.

Beyond protein, lipid, and DNA damage, oxidative stress leads to cholesterol oxidation, generating oxysterols—biologically active cholesterol metabolites that play complex roles in prostate cancer progression. Oxysterols such as 27-hydroxycholesterol (27-HC) and 25-hydroxycholesterol (25-HC) function as endogenous ligands for liver X receptors (LXRs), which act as tumor suppressors in prostate cancer by inducing cell cycle arrest and limiting inflammation through inhibition of *IL-6*, *COX-2*, and *iNOS* expression [40]. Loss of the oxysterol-sulfating enzyme *SULT2B1b* in castration-resistant prostate cancer leads to enhanced proliferation, migration, and epithelial–mesenchymal transition, while oxysterol production normally limits invasiveness [41]. Notably, *27-HC* downregulates DNA damage repair pathways and induces “BRCAness” in prostate cancer cells, with higher *CYP27A1* expression correlating with favorable outcomes [42]. Furthermore, oxysterols influence ferroptosis susceptibility through membrane lipid remodeling: *25-HC* suppresses *GPX4* expression while upregulating lipid peroxidation-promoting enzymes *CYB5R1* and *POR* [43]. Oxysterols also modulate the tumor immune microenvironment by dampening anti-tumor immune responses and promoting pro-inflammatory states [44], creating multiple therapeutic vulnerabilities at the intersection of oxidative stress, ferroptosis, and immune regulation.

## 3. Lipid Metabolism and Ferroptosis Execution

### 3.1. PUFA-Phospholipid Biosynthesis

*ACSL4* exhibits unique selectivity for long-chain PUFAs (arachidonic and adrenic acid), converting them into acyl-CoA thioesters for phospholipid incorporation [14]. This substrate specificity enriches cellular membranes with highly oxidizable lipids, creating ferroptosis vulnerability. In PCa, *ACSL4* expression correlates with metastatic potential, linking aggressive phenotypes to targetable metabolic dependencies [14]. *LPCAT3* completes ferroptosis-susceptible phospholipid synthesis by incorporating *ACSL4*-activated PUFA-CoAs at the sn-2 position of lysophospholipids [45]. Phospholipids containing PUFAs at both sn-1 and sn-2 positions (di-PUFA species) exhibit several-fold higher peroxidation susceptibility [45]. Phosphatidylethanolamine species with two arachidonic or adrenic acid chains represent the most vulnerable membrane components, with concentrations strongly correlating with ferroptosis sensitivity.

### 3.2. Phospholipid Remodeling: Defense Mechanism

#### 3.2.1. MBOAT1/2 Family

*MBOAT1* and *MBOAT2* (membrane-bound O-acyltransferase 1/2) represent a distinct ferroptosis defense mechanism operating through upstream phospholipid remodeling [19]. Unlike *GPX4* and *FSP1* that detoxify lipid peroxides after formation [15,16], *MBOAT1/2* prevent accumulation of ferroptosis-susceptible phospholipids. These enzymes exhibit selectivity for monounsaturated fatty acids (MUFAs) and saturated fatty acids (SFAs) over polyunsaturated fatty acids (PUFAs) [19], contrasting with *LPCAT3*’s PUFA preference [45]. By incorporating MUFAs and SFAs into phospholipids, *MBOAT1/2* remodel cellular membranes away from highly oxidizable PUFA-containing species, fundamentally decreasing ferroptosis susceptibility independent of antioxidant capacity [19].

#### 3.2.2. Sex Hormone Regulation

Sex hormone regulation of *MBOAT1/2* has critical implications for hormone-dependent cancers. *MBOAT1* responds to estrogen receptor signaling, whereas *MBOAT2* is transcriptionally regulated by androgen receptor [19]. In hormone-sensitive PCa, AR-driven *MBOAT2* expression maintains a phospholipid profile enriched in MUFAs and SFAs, conferring ferroptosis resistance [19]. ADT suppresses *MBOAT2* expression, increasing PUFA incorporation and creating a therapeutic vulnerability window for ferroptosis induction [19]. However, in CRPC where AR signaling persists through AR amplification or splice variants [46,47], *MBOAT2* levels remain elevated, contributing to ferroptosis resistance through continued lipid remodeling [19].

### 3.3. Iron-Catalyzed Lipid Peroxidation

The labile iron pool comprises redox-active Fe^2+^/Fe^3+^ that provides iron for biological functions while driving ferroptosis when excessive [48]. Transferrin receptor 1 (TfR1) mediates iron import [49], and cancer cells frequently upregulate *TfR1* to meet proliferative demands, inadvertently expanding the ferroptosis-vulnerable labile pool. Ferritin stores iron safely, while ferritinophagy (via NCOA4) mobilizes stored iron [50], creating a dynamic balance that determines ferroptosis susceptibility. Ferrous iron catalyzes lipid hydroperoxide decomposition through Fenton-like reactions, generating lipid alkoxyl and peroxyl radicals that abstract hydrogen from adjacent PUFAs, perpetuating chain reactions [51]. This catalytic cycle enables small amounts of iron to cause extensive peroxidation when *GPX4* is inhibited. Uncontrolled lipid peroxidation severely disrupts membrane structure. Polar hydroperoxide groups distort the bilayer, creating permeability defects [38]. Advanced peroxidation causes pore formation, uncontrolled ion flux, and plasma membrane rupture. Unlike coordinated apoptosis, ferroptotic death is chaotic and inflammatory, releasing damage-associated molecular patterns that trigger immune responses [11].

### 3.4. Reactive Aldehydes: Secondary Messengers

Lipid peroxidation generates reactive aldehydes, such as 4-hydroxynonenal (4-HNE), malondialdehyde (MDA), and acrolein, which modify proteins, lipids, and DNA [52,53]. These electrophiles consume GSH through conjugation reactions, further depleting the antioxidant defenses and creating a vicious cycle. *ALDH7A1* detoxifies these aldehydes through NAD^+^-dependent oxidation to less toxic carboxylic acids [17]. The balance between aldehyde generation and detoxification determines whether it contributes to signaling or toxicity. During ferroptosis, the overwhelming production exceeds the detoxification capacity, amplifies cellular damage, and accelerates death.

## 4. Pro-Oxidant Therapeutic Strategies

### 4.1. Menadione-Based Pro-Oxidant Therapy: A Novel Mechanistic Approach

#### 4.1.1. VPS34 Targeting and Triaptosis

Menadione sodium bisulfite (MSB), a vitamin K3 precursor, demonstrates potent efficacy in preclinical PCa models through oxidative targeting of *VPS34* (*PIK3C3*), a master regulator of endosomal function [54]. *MSB*-induced redox cycling generates ROS that selectively oxidize critical cysteine residues in *VPS34*, abolishing its kinase activity and disrupting endosomal phosphatidylinositol 3-phosphate (PI3P) generation [54]. Cancer cells’ heightened basal oxidative stress makes them preferentially susceptible compared to normal cells [54].

This *VPS34* oxidation triggers ‘triaptosis,’ a distinct regulated cell death characterized by catastrophic endosomal dysfunction [55]. Unlike ferroptosis (lipid peroxidation) or apoptosis (caspase-mediated), triaptosis centers on *PI3P* depletion and progressive endosomal swelling without caspase activation [55]. Triaptosis circumvents common resistance mechanisms: cells resistant to apoptosis (*BCL-2* overexpression, *TP53* mutations) or ferroptosis (elevated *GPX4/FSP1*) remain vulnerable [55]. This *VPS34*-targeting strategy exploits orthogonal vulnerabilities, enabling rational combination approaches.

#### 4.1.2. Proteome-Wide Oxidative Effects

Redox proteomics revealed that menadione preferentially oxidizes translation machinery, particularly eukaryotic initiation factors (EIF2, EIF6) and elongation factor 2 (EEF2) [36]. This selective oxidation disrupts protein synthesis and prevents antioxidant enzyme production, creating a positive feedback loop that amplifies oxidative damage [35,36]. The specificity for translation factors suggests pro-oxidants exert therapeutic effects through targeted protein network disruption rather than indiscriminate oxidation, potentially explaining *MSB*’s tumor selectivity.

### 4.2. Ferroptosis Induction Strategies

#### 4.2.1. GPX4 Inhibition

*RSL3* and *ML162* covalently inhibit *GPX4*, abolishing lipid hydroperoxide reduction [56]. In PCa, enzalutamide-resistant CRPC cells demonstrate *RSL3* susceptibility, with ferrostatin-1 reversal confirming ferroptosis as the death mechanism [57]. However, hypoxia induces resistance by suppressing *ACSL4/LPCAT3* expression and promoting lipid droplet accumulation, sequestering PUFAs from membrane phospholipids [56]. Genetic alterations also determine *GPX4*-dependent ferroptosis sensitivity. *PTEN* loss, occurring in approximately 20% of primary PCa and 50% of CRPC, promotes *GPX4* transcription independent of *PI3K/AKT* signaling, resulting in elevated *GPX4* protein levels and reduced cellular ROS [58]. Consequently, *PTEN*-null PCa cells (PC3, LNCaP) exhibit marked resistance to erastin-induced ferroptosis compared to *PTEN* wild-type cells (DU145). Importantly, *GPX4* knockdown restores ferroptosis sensitivity in *PTEN*-deficient cells, indicating that *GPX4* inhibitors are essential therapeutic components for *PTEN*-loss tumors [58]. *GPX4* inhibitor efficacy varies with cellular context, necessitating combination approaches targeting multiple pathways.

#### 4.2.2. System Xc^−^ Blockade and Glutamine Metabolism

System Xc^−^ inhibitors (erastin, sulfasalazine, sorafenib) block *SLC7A11*-mediated cystine import, depleting GSH and compromising *GPX4* activity. In PCa, AR regulates *SLC7A11* expression, creating therapeutic opportunities: ADT suppresses *SLC7A11*, sensitizing cells to system Xc^−^ inhibitors during androgen deprivation [19,47].

Glutamine metabolism provides alternative GSH synthesis, conferring ferroptosis resistance. YAP1, elevated in CRPC, upregulates *SLC1A5* (glutamine transporter) and *GLS1* (glutaminase), driving glutamine-to-glutamate conversion for GSH synthesis [59]. This *YAP1*-glutamine-GSH axis suppresses *RSL3*-induced ferroptosis. Combined *SLC1A5* (V-9302) or *GLS1* (CB-839) inhibition with ferroptosis inducers overcomes this resistance.

However, multiple resistance mechanisms limit System Xc^−^ inhibitor efficacy. Circular RNAs confer ferroptosis resistance through *SLC7A11* upregulation: *circCNOT6L* promotes *SLC7A11* alternative splicing via the miR-143-5p/SRSF2 axis, driving both ferroptosis resistance and metastasis [30], while *circATP2C1* upregulates *SLC7A11* through miR-654-3p sequestration [31]. Transcription factor *HOXA13* directly activates both System Xc^−^ subunits (SLC7A11 and SLC3A2), and *HOXA13* knockdown combined with ferroptosis inducers significantly suppresses tumor growth in vivo [29]. These findings suggest that targeting upstream regulators of *SLC7A11*—including circRNAs and transcription factors—may overcome resistance to conventional System Xc^−^ inhibitors. Notably, combined circCNOT6L silencing with erastin effectively suppresses tumor growth in patient-derived organoid models [30], and *circATP2C1* knockdown synergizes with erastin in xenograft models [31].

#### 4.2.3. FSP1 Inhibition

*FSP1* inhibitors (iFSP1) target the *GPX4*-independent *CoQ10* pathway [16]. Combined *GPX4-FSP1* inhibition achieves synthetic lethality: cells tolerate single-pathway loss but succumb to dual blockade. This approach demonstrated enhanced efficacy in preclinical models, suggesting clinical potential for CRPC with multiple coexisting resistance mechanisms.

### 4.3. Rational Combination Strategies: A Mechanistic Framework

Redundant ferroptosis defense systems necessitate rational combination strategies targeting multiple protective pathways. We categorize combinations into three mechanistic frameworks: (1) vertical inhibition targeting multiple steps within single pathways, (2) horizontal inhibition achieving synthetic lethality across parallel pathways, and (3) vulnerability induction creating exploitable dependencies through therapeutic perturbation. This framework enables rational selection of combination partners, dosing schedules, and treatment sequences based on tumor-specific defense configurations.

#### 4.3.1. Vertical Inhibition: Multi-Step Targeting of Single Defense Pathways

**Figure 2:**
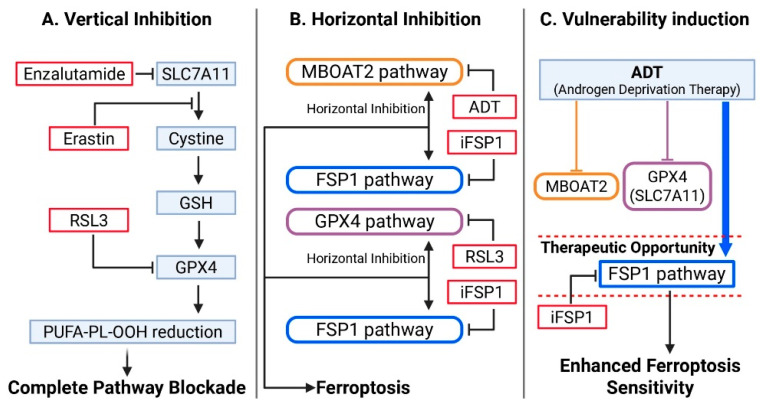
Strategic frameworks for ferroptosis-based combination therapy in prostate cancer. (**A**) Vertical inhibition: multi-step targeting of a single defense pathway (e.g., enzalutamide blocks AR-mediated *SLC7A11* transcription, erastin inhibits *SLC7A11* transport function, *RSL3* inactivates GPX4), achieving complete pathway blockade. (**B**) Horizontal inhibition: simultaneous targeting of parallel, mechanistically independent defense systems (e.g., ADT suppresses *MBOAT2* pathway, *iFSP1* blocks *FSP1-CoQ10* system, *RSL3* inhibits *GPX4* pathway), creating synthetic lethality. (**C**) Vulnerability induction: ADT suppresses AR-driven *MBOAT2* and *GPX4* (via *SLC7A11*) expression while cells maintain *FSP1* pathway dependency, creating a therapeutic window for *FSP1* inhibitor-mediated ferroptosis. This framework enables rational selection of combination partners based on tumor-specific defense configurations. Arrows indicate; solid lines represent direct inhibition/suppression, dashed lines represent indirect effects or pathway dependencies.

Vertical inhibition targets multiple dependent steps within single defense pathways, overwhelming pathway capacity through redundant blockade at serial checkpoints (Figure 2A). This strategy prevents compensation by attacking mechanistically distinct regulatory nodes (transcription, protein stability, catalytic function) within pathways.

The canonical example combines system Xc^−^ and *GPX4* inhibition: erastin blocks cystine import via *SLC7A11*, depleting GSH substrate for *GPX4*, while *RSL3* covalently inactivates *GPX4*’s catalytic selenocysteine [56]. This dual blockade achieves complete pathway shutdown unattainable by either agent alone.

The enzalutamide–erastin combination reveals how vertical inhibition circumvents adaptive resistance [60]. Enzalutamide suppresses AR-mediated *SLC7A11* transcription, but cells compensate through post-translational stabilization by downregulating *NEDD4L* (E3 ubiquitin ligase) [60]. This transcriptional suppression offset by protein stabilization limits ferroptosis sensitization with enzalutamide monotherapy. Erastin circumvents this by directly blocking *SLC7A11* transport function regardless of protein abundance, achieving pathway shutdown despite compensatory mechanisms [60]. This illustrates the critical principle: vertical inhibition succeeds by targeting distinct regulatory nodes, creating threshold effects where pathways catastrophically fail.

#### 4.3.2. Horizontal Inhibition: Synthetic Lethality Across Parallel Defense Pathways

Horizontal inhibition exploits architectural redundancy by simultaneously targeting parallel, mechanistically independent defense systems, creating synthetic lethality where loss of either pathway alone is tolerable but combined loss is lethal (Figure 2B). This strategy circumvents compensatory resistance mechanisms that enable cells to survive single-pathway inhibition.

*RSL3* + *iFSP1* exemplifies this approach [15,16]. *GPX4* reduces phospholipid hydroperoxides using GSH [15], while *FSP1* reduces *CoQ10* to trap lipid peroxyl radicals independently of GSH [16]. Cancer cells tolerate single-pathway loss through compensatory upregulation, but simultaneous dual inhibition creates catastrophic lipid peroxidation [16].

ADT + *FSP1* inhibitors target upstream (*MBOAT2*) and downstream (*FSP1*) defenses [16,19]. ADT suppresses AR-driven *MBOAT2*, increasing PUFA-PL accumulation [19], while cells compensate through *FSP1*-mediated radical scavenging. *FSP1* inhibition removes this parallel defense, rendering PUFA-enriched membranes vulnerable to peroxidation. This approach bypasses AR-V7-mediated constitutive *SLC7A11* expression in CRPC [47], circumventing rather than confronting the *GPX4*-GSH axis resistance.

#### 4.3.3. Vulnerability Induction: Creating Dependencies Through Therapeutic Perturbation

Vulnerability induction strategies differ fundamentally from direct inhibition by remodeling cellular metabolic state to enhance ferroptosis susceptibility through sequential therapeutic perturbation (Figure 2C). The initial therapy establishes a vulnerable state subsequently exploited by ferroptosis inducers, requiring temporal optimization rather than simultaneous administration.

ADT represents the most therapeutically relevant vulnerability induction for PCa [19,61]. AR target gene suppression reduces *SLC7A11* (decreasing cystine import and GSH synthesis) and *MBOAT2* (increasing PUFA-PL content), creating a ferroptosis-permissive state [19,47]. Additional metabolic rewiring—altered lipid droplet dynamics, iron regulatory protein expression changes, and mitochondrial perturbations—further sensitizes cells [61]. These changes establish a landscape where cells become extremely sensitive to ferroptosis inducers administered during this therapeutic window. Lipidomic profiling reveals ADT-induced lipid remodeling peaks at specific temporal windows, creating time-dependent vulnerability exploitable through sequential *FSP1* inhibitor administration [61].

#### 4.3.4. Clinical Translation: Framework-Guided Combination Design

This framework enables rational, biomarker-guided combination design tailored to tumor-specific resistance mechanisms. High *GPX4*/low *FSP1* tumors: vertical inhibition (erastin + *RSL3*). Balanced *GPX4/FSP1* co-expression: horizontal inhibition (*RSL3* + *iFSP1*) [15,16]. Hormone-sensitive disease: vulnerability induction through ADT followed by sequential ferroptosis inducers, exploiting metabolic perturbation windows [19,61]. AR-V7-positive CRPC with constitutive *SLC7A11*: horizontal targeting bypassing *GPX4*-GSH axis (*FSP1* inhibitors or *MBOAT2*-targeting strategies) [47].

This framework also informs clinical trial design. Vertical inhibition trials focus on dose optimization for complete pathway shutdown. Horizontal inhibition trials require biomarker stratification to identify active parallel defenses. Vulnerability induction trials incorporate pharmacodynamic monitoring (lipidomic profiling, GSH measurements) to identify optimal sequential dosing timing rather than assuming concurrent superiority.

#### 4.3.5. Immunotherapy Integration: A Multi-Mechanism Combination

Ferroptosis induces immunogenic cell death characterized by DAMP release (HMGB1, ATP, calreticulin), priming anti-tumor immunity [62,63]. In immunologically “cold” PCa, ferroptosis transforms the tumor microenvironment (TME) from immune-excluded to immune-infiltrated through dendritic cell activation and M1 macrophage polarization [62,64]. Combined ferroptosis-checkpoint inhibitor therapy exploits this immune activation. Peptide-based *GPX4*-targeting approaches enable spatiotemporal control through light-activated ferroptosis induction coupled with cGAS-STING pathway activation [65].

## 5. Androgen Receptor-Mediated Redox Regulation

The relationship between androgen receptor signaling and ferroptosis susceptibility exemplifies cancer evolution under therapeutic pressure. This section traces AR’s evolutionary trajectory from hormone-sensitive disease, where AR coordinates proliferation and ferroptosis defenses, through castration-resistant adaptations preserving these defenses despite androgen deprivation. Understanding this progression reveals why single-agent therapies often fail and provides a framework for resistance stage-specific combination strategies.

### 5.1. AR Signaling in Prostate Cancer

#### 5.1.1. AR Structure and Function

The AR, a ligand-activated transcription factor, governs PCa through genomic and non-genomic mechanisms [66]. Upon androgen binding, AR translocates to the nucleus, binds androgen response elements (AREs), and drives transcription of genes controlling proliferation, survival, and metabolism. This transcriptional program extends beyond classical proliferative targets to encompass redox balance pathways, including ferroptosis defense genes (*SLC7A11*, *MBOAT2*).

This AR-centered orchestration explains PCa treatment resistance evolution (Figure 1). In hormone-sensitive disease, AR links androgen availability to proliferation and ferroptosis defenses. ADT selective pressure drives adaptation through AR-V7 splice variants (ligand-independent transcription) [47], AR amplification (hypersensitivity to residual androgens), and AR-independent survival programs like *SPOP-JMJD6* bypass [64]. Understanding this progression is essential for developing stage-specific therapeutic interventions that match combination strategies to the underlying resistance mechanisms.

#### 5.1.2. AR and Metabolic Reprogramming

The AR coordinates metabolic reprogramming supporting both growth and redox homeostasis in PCa. AR-driven transcriptional programs regulate lipid synthesis, mitochondrial function, and redox balance, enabling PCa cells to tolerate elevated oxidative stress inherent to malignant transformation [66]. Consequently, ADT disrupts these interconnected metabolic pathways, creating exploitable vulnerabilities for pro-oxidant or ferroptosis-inducing therapies. This metabolic disruption represents a critical therapeutic window for combination strategies targeting cancer cell redox homeostasis.

### 5.2. AR-Regulated Ferroptosis Defense Mechanisms

#### 5.2.1. Direct Transcriptional Targets

The AR directly regulates three ferroptosis defense genes in PCa (Table 2). MBOAT2-mediated phospholipid remodeling reduces PUFA-PL substrate availability [19]. *SLC7A11* maintains cystine import and GSH synthesis, supporting *GPX4* activity [47]. *PEX10* contributes to peroxisomal integrity and lipid homeostasis [66]. Notably, enzalutamide paradoxically increases *SLC7A11* protein levels by downregulating *NEDD4L*, an E3 ubiquitin ligase targeting *SLC7A11* for degradation [60]. This post-translational stabilization mechanism explains limited ferroptosis sensitization with AR inhibitor monotherapy and provides rationale for combining AR antagonists with system Xc^−^ blockers like erastin.

#### 5.2.2. Mechanism of Ferroptosis Resistance

The AR-regulated defense mechanisms converge on two fundamental strategies: reducing substrate availability and enhancing antioxidant capacity. Through *MBOAT2*-mediated phospholipid remodeling, AR signaling reduces PUFA-PL content, the primary ferroptosis substrate, thereby creating a lipid membrane composition inherently resistant to peroxidation [19]. This compositional shift toward saturated and monounsaturated phospholipids enhances membrane stability and reduces susceptibility to lipid peroxidation chain reactions. Simultaneously, AR-driven *SLC7A11* expression maintains robust cystine import, GSH synthesis, and *GPX4* activity, enabling efficient neutralization of lipid peroxides that do form [47]. This dual-pronged defense, combining substrate reduction with enhanced antioxidant capacity, explains the remarkable ferroptosis resistance observed in androgen-replete PCa cells and highlights why ADT disruption of these pathways creates therapeutic vulnerability windows.

### 5.3. ADT and Ferroptosis Sensitization

#### 5.3.1. Metabolic Consequences of ADT

ADT disrupts multiple AR-controlled metabolic pathways, creating a transient window of ferroptosis vulnerability. *MBOAT2* downregulation shifts phospholipid composition toward greater PUFA content, increasing the pool of oxidizable substrates [19]. Simultaneously, *SLC7A11* suppression reduces cystine import and GSH synthesis, compromising *GPX4*-mediated lipid peroxide detoxification [47]. These coordinated metabolic perturbations converge to create oxidative stress that exceeds cellular compensatory capacity, rendering cells vulnerable to ferroptosis induction.

#### 5.3.2. Therapeutic Window Creation

The loss of AR-mediated protection creates a critical therapeutic window for ferroptosis induction. Maximal sensitization likely occurs during the early ADT response, before adaptive resistance mechanisms engage. During this period, cells experience simultaneous depletion of multiple ferroptosis defenses while lacking compensatory survival pathways. Sequential or concurrent administration of ferroptosis inducers during this window may achieve optimal therapeutic efficacy, exploiting the metabolic vulnerability created by androgen deprivation. However, this window proves transient. CRPC emergence reflects successful adaptation through AR amplification, AR-V7 expression enabling ligand-independent signaling, or activation of alternative survival pathways [47,59]. AR-V7-mediated *SLC7A11* upregulation restores GSH-*GPX4* activity, while persistent *MBOAT2* expression maintains lipid remodeling defenses. In *SPOP*-mutated subsets, *JMJD6*-driven ferroptosis resistance provides complete independence from AR signaling [67]. Effective therapeutic intervention requires matching treatment strategies to the specific resistance stage, transforming patient stratification from empirical biomarker testing to mechanistically rational selection based on the underlying resistance biology.

### 5.4. Combination Therapy Strategies

#### 5.4.1. AR Antagonists + Ferroptosis Inducers

Several evidence-based combination strategies targeting the AR-ferroptosis crosstalk have shown promising preclinical results. The combination of enzalutamide with erastin addresses a paradoxical resistance mechanism wherein enzalutamide-induced *NEDD4L* downregulation stabilizes *SLC7A11* protein [60]. Erastin blocks system Xc^−^ function, preventing cystine import despite elevated *SLC7A11* levels, thereby restoring ferroptosis sensitivity [60]. Another rational approach combines AR-targeting proteolysis-targeting chimeras (PROTACs) with *GPX4* inhibitors. PROTACs degrade both AR-FL and AR-V7, eliminating transcriptional support for ferroptosis defenses [46]. When combined with *RSL3*-mediated *GPX4* inhibition, this strategy achieved synthetic lethality in AR-V7-expressing CRPC cells [48]. Additionally, combining ADT with *FSP1* inhibitors targets parallel defense pathways: ADT suppresses the *SLC7A11*-GSH-*GPX4* axis while *FSP1* inhibition blocks *CoQ10*-mediated lipid peroxyl radical trapping, creating dual vulnerability [66]. Beyond conventional combination approaches, novel therapeutic modalities targeting *SLC7A11* degradation have emerged. The recombinant *CCDC7_241__aa_* protein, derived from the chimeric circular RNA *CCDC7_19–13_*, promotes *TRIM21*-mediated *SLC7A11* ubiquitination and demonstrates synergistic effects with both docetaxel and enzalutamide across AR-negative (PC3) and AR-positive (LNCaP, VCaP, 22RV1) PCa cell lines [32]. In patient-derived xenograft models of CRPC, *CCDC7_241__aa_* effectively suppresses tumor growth without apparent toxicity, suggesting clinical translatability [32].

#### 5.4.2. Novel Dual-Function Agents

Several compounds demonstrate simultaneous AR inhibition and ferroptosis induction, representing pharmacologically elegant single-agent approaches. Darolutamide, a second-generation AR antagonist approved for CRPC, may possess pro-ferroptotic properties beyond AR blockade [66]. Erastin exhibits dual activity by inhibiting both system Xc^−^ and displaying off-target effects on AR signaling. Structure–activity relationship studies exploring AR antagonist scaffolds with ferroptosis-inducing modifications may yield optimized dual-function molecules with improved therapeutic indices.

#### 5.4.3. Overcoming CRPC

CRPC emergence through AR amplification, AR-V7 expression, or AR-independent pathways restores ferroptosis resistance despite initial ADT sensitization. AR-V7-positive CRPC maintains persistent *SLC7A11* expression through constitutive transcriptional activation, necessitating *FSP1* inhibition to bypass the *GPX4*-GSH axis [16,47]. *SPOP*-mutated tumors achieve complete AR independence through *JMJD6*-driven ferroptosis resistance mediated by *ATF4*-dependent enhancement of GSH metabolism [67]. These tumors require combined *JMJD6* inhibition and ferroptosis induction [67]. This evolutionary framework transforms therapeutic selection from empirical approaches to mechanistically rational strategies matched to specific resistance stages. Effective CRPC treatment requires biomarker-guided identification of the dominant resistance mechanism, enabling precision targeting of the underlying ferroptosis defense pathway.

## 6. Clinical Translation and Future Perspectives

### 6.1. Redox Biomarkers for Patient Stratification

Effective clinical implementation requires predictive biomarkers. Oxidative stress markers (GSH/GSSG ratio, lipid peroxidation products [52,53], 8-oxo-dG [39]) provide complementary assessments of redox balance and oxidative damage. Molecular signatures guide therapeutic selection: *ACSL4/LPCAT3* expression predicts ferroptosis susceptibility [14,45], *GPX4/FSP1* ratios determine dominant defense pathways [16,28], AR-V7 status indicates constitutive *SLC7A11* activity requiring *FSP1*-targeting approaches [47], and *SPOP* mutations signal *JMJD6*-driven resistance [67]. Lipidomic profiling quantifies PUFA-PL substrate pools [61], with ADT-induced remodeling creating temporal vulnerability windows for optimal ferroptosis inducer timing [61]. Integrating these molecular, metabolic, and genomic biomarkers enables patient stratification based on specific vulnerability profiles (Table 3). Clinical implementation of these biomarkers requires consideration of practical feasibility. Oxidative stress markers (GSH/GSSG ratio, 4-HNE, MDA) and serum iron parameters are readily accessible through standard clinical laboratories using spectrophotometry or ELISA-based assays. However, comprehensive PUFA-PL profiling necessitates specialized lipidomics platforms with liquid chromatography–tandem mass spectrometry (LC-MS/MS) capabilities, currently available only in research or tertiary care centers [61]. Molecular signatures (*ACSL4*, *LPCAT3*, *GPX4*, *FSP1*) require tissue biopsies for immunohistochemistry or quantitative PCR analysis, though emerging liquid biopsy technologies enable minimally invasive monitoring of AR-V7 status and genomic alterations through circulating tumor cells [47]. Recent studies have identified additional biomarkers for ferroptosis therapy stratification. *PTEN* status critically determines ferroptosis susceptibility: *PTEN* wild-type tumors respond to System Xc^−^ inhibitors alone, whereas *PTEN*-null tumors require *GPX4* inhibitor combinations due to elevated *GPX4* expression [58]. Circular RNA expression profiles offer prognostic value; high *circATP2C1* expression correlates with poor overall survival, elevated Gleason score, and metastasis, potentially serving as a predictive biomarker for ferroptosis therapy resistance [31]. Similarly, low *CCDC7_19–13_* expression independently predicts poor prognosis in advanced and recurrent PCa and may identify patients who would benefit from *SLC7A11*-targeted therapies [32]. *HOXA13* overexpression marks metastatic tumors with enhanced System Xc^−^ activity that may require *HOXA13*-targeted intervention combined with ferroptosis inducers [29]. A tiered diagnostic approach—combining readily available markers for initial screening with advanced lipidomic and genomic profiling for high-risk patients—may optimize cost-effectiveness while maintaining predictive accuracy for ferroptosis-based therapeutic stratification.

### 6.2. Challenges in Clinical Translation

Achieving cancer-selective toxicity while sparing normal tissues remains the fundamental challenge. Tumor-targeted delivery using PSMA-conjugated nanoparticles [64], prodrug approaches exploiting tumor-enriched enzymes, and temporal dosing during ADT-induced sensitization windows [19,47] can enhance selectivity. Resistance mechanisms include *NRF2*-mediated transcriptional upregulation of antioxidant programs [10], lipid droplet accumulation sequestering PUFAs [56], enhanced glutamine metabolism supporting GSH synthesis [59], and defense pathway redundancy [16,19,28]. Rational combinations targeting multiple mechanisms simultaneously can prevent adaptive escape. Many first-generation ferroptosis inducers exhibit suboptimal pharmacokinetics including poor solubility and limited tumor penetration [56]. Nanoparticle-based delivery systems address these challenges through enhanced solubility, prolonged circulation, and active PSMA-targeting [64]. However, clinical translation requires coordinated medicinal chemistry programs, biomarker validation, and adaptive trial designs incorporating pharmacodynamic endpoints [60,66].

### 6.3. Clinical Translation Pathway

Despite substantial preclinical advances, direct ferroptosis-inducing strategies remain absent from current PCa clinical trials, reflecting pharmacokinetic limitations of first-generation inducers and lack of validated biomarkers [56]. Pro-oxidant approaches including menadione sodium bisulfite (MSB) show promise through *VPS34* oxidation and triaptosis induction [54,55]. Near-term clinical opportunities may arise from rational combinations integrating AR antagonists with ferroptosis inducers [60,66] or coupling oxidative stress induction with immunotherapy [62,65]. Early phase trials should incorporate comprehensive biomarker assessments to guide patient selection and subsequent development.

### 6.4. Future Research Directions

PCa molecular heterogeneity demands biomarker-guided therapeutic strategies [66,67]. Clinical integration requires validated algorithms integrating *ACSL4/LPCAT3* expression, *GPX4/FSP1* ratios, AR-V7 status, and lipidomic profiles [14,45,47,61] to generate quantitative ferroptosis sensitivity scores. Real-time monitoring through liquid biopsies should enable adaptive therapy adjustments. Novel modalities including photodynamic and sonodynamic therapies offer spatiotemporal ROS control, while CRISPR-based strategies targeting ferroptosis defense genes demonstrate preclinical potential. Ferroptosis-induced immunogenic cell death [62,63] enables synergy with checkpoint inhibitors, potentially transforming immunologically “cold” prostate tumors. Beyond PCa, ferroptosis-targeting strategies extend to hormone-dependent cancers (breast, ovarian) exhibiting estrogen receptor-mediated *MBOAT1* regulation [19], melanoma with high *ACSL4* expression [14], and lipid-rich clear cell renal carcinoma.

### 6.5. Conclusions

This review establishes ferroptosis targeting in prostate cancer as a precision medicine paradigm exploiting two fundamental principles. First, “robustness through redundancy”: cancer cells survive through multiple parallel defense pathways (*GPX4*-GSH, *FSP1-CoQ10*, *ALDH7A1*, *MBOAT1/2*) [16,19,28], necessitating multi-pathway targeting for synthetic lethality. Second, the “evolutionary arms race” between AR signaling and ferroptosis resistance: hormone-sensitive cells depend on AR-coordinated defenses (*SLC7A11*, *MBOAT2*) [19,47], ADT creates transient vulnerability windows, but CRPC emerges through progressive adaptations—AR-V7 constitutive activity [47], AR amplification, and JMJD6-ATF4 bypass [67]—that restore oxidative resistance. This framework establishes three rational combination strategies: vertical inhibition targeting multiple pathway steps, horizontal inhibition achieving synthetic lethality across parallel defenses, and vulnerability induction creating exploitable dependencies [19,47,61]. Successful therapy requires stage-specific matching: hormone-sensitive disease benefits from ADT–ferroptosis combinations exploiting *SLC7A11/MBOAT2* suppression [19,47]; AR-V7-positive CRPC necessitates *FSP1* inhibition bypassing the *GPX4*-GSH axis [16,47]; *SPOP*-mutated tumors require *JMJD6* inhibitor integration [67]. Novel approaches including menadione-based *VPS34* targeting induce triaptosis [54,55], while ferroptosis-induced immunogenic cell death enables checkpoint inhibitor synergy [62,63]. Clinical translation requires validated biomarkers (*ACSL4/LPCAT3*, *GPX4/FSP1* ratios, AR-V7 status, lipidomic profiles) [14,45,47,61] for patient stratification, next-generation inducers with improved pharmacokinetics [56], tumor-targeted delivery [64], and rational combination trials [60,66]. Emerging evidence reveals additional layers of *SLC7A11* regulation—including circular RNA-mediated post-transcriptional control [30,31], transcription factor *HOXA13* [29], and *TRIM21*-mediated ubiquitination [32]—that expand therapeutic targeting opportunities. Furthermore, genetic context determines ferroptosis sensitivity, as *PTEN*-loss tumors exhibit *GPX4*-dependent resistance requiring tailored combination strategies [58].

This represents a fundamental reconceptualization: ferroptosis vulnerability as an evolutionary battleground shaped by redundant defenses and AR-mediated adaptations, providing a framework for stage-specific, biomarker-guided interventions that exploit the architectural fragility beneath cancer’s oxidative robustness.

## Figures and Tables

**Table 1:** Distinguishing Features of Ferroptosis from Other Cell Death Pathways. Ferroptosis is uniquely characterized by iron-dependent lipid peroxidation of PUFA-containing phospholipids, distinguishing it from caspase-dependent apoptosis and RIPK-mediated necroptosis. PUFA-PL, polyunsaturated fatty acid-containing phospholipids.

Feature	Ferroptosis	Apoptosis	Necroptosis	Refs.
Iron dependency	Required	No	No	[11]
Lipid peroxidation	Essential	No	No	[11,12]
Caspase activation	No	Yes	No	[12]
Key regulators	*GPX4*, *FSP1*, *ACSL4*	Caspases, Bcl-2	*RIPK1/3*, *MLKL*	[13,15,16]
Mitochondria	Shrinkage	Swelling	Swelling	[12]
PUFA-PL requirement	Yes	No	No	[13,14]

**Table 2:** AR directly regulates ferroptosis defense genes in PCa. MBOAT2 reduces substrate availability through lipid remodeling. *SLC7A11* maintains *GPX4* function via GSH synthesis. *PEX10* supports peroxisomal lipid metabolism. ADT suppression creates ferroptosis vulnerability, while AR-V7 maintains constitutive expression in CRPC, contributing to resistance.

Gene	Function	AR Regulation	Defense Mechanism	ADT Impact	CRPC Resistance	Refs.
***MBOAT2***	Lysophospholipid acyltransferase	Direct transcriptional activation	Incorporates MUFA/SFA into phospholipids, reducing PUFA-PL content	Suppression increases PUFA-PL and ferroptosis sensitivity	AR-V7 maintains expression; AR amplification restores activity	[19]
***SLC7A11***	Cystine-glutamate antiporter	Direct transcriptional activation via AREs	Maintains cystine import for GSH synthesis and GPX4 activity	Suppression depletes GSH, sensitizing to ferroptosis	AR-V7 drives constitutive expression; enzalutamide stabilizes protein via NEDD4L suppression	[47,60]
***PEX10***	Peroxisomal biogenesis factor	AR-regulated expression	Supports peroxisomal β-oxidation, prevents oxidation-prone lipid accumulation	Suppression compromises peroxisomal function	Restoration through persistent AR activity	[66]

**Table 3:** Integrated biomarker panel for precision ferroptosis therapy stratification. Oxidative stress markers establish baseline vulnerability. Molecular signatures (*ACSL4/LPCAT3*, *GPX4/FSP1* ratio, AR-V7, *SPOP*) predict intrinsic sensitivity and resistance mechanisms. Lipidomic profiling quantifies substrate pools, enabling optimal timing during ADT-created vulnerability windows. Iron homeostasis reflects Fenton reaction capacity.

Category	Biomarker	Biological Meaning	Clinical Application	Refs.
**Oxidative Stress**	GSH/GSSG ratio	Redox balance, antioxidant capacity	Baseline vulnerability	[27]
	4-HNE, MDA	Lipid peroxidation products	Oxidative damage status	[52,53]
	8-oxo-dG	DNA oxidation	Disease severity	[39]
**Sensitivity Signature**	*ACSL4*, *LPCAT3*	PUFA-PL synthesis	Intrinsic susceptibility	[14,45]
	*GPX4/FSP1* ratio	Defense pathway dominance	Guide inhibitor choice	[16,28]
	AR-V7	Constitutive defense	*FSP1* inhibitor need	[47]
	*SPOP* mutations	AR-independent resistance	*JMJD6* inhibitor need	[67]
**Metabolic**	PUFA-PL abundance	Substrate availability	ADT timing optimization	[61]
	Serum iron	Fenton reaction capacity	Iron status	[48,49,50]

## Data Availability

No new data were created or analyzed in this study. Data sharing is not applicable to this article.

## Abbreviations

The following abbreviations are used in this manuscript:

4-HNE	4-Hydroxynonenal
8-oxo-dG	8-oxo-deoxyguanosine
ACSL4	Acyl-CoA Synthetase Long-Chain Family Member 4
ADT	Androgen Deprivation Therapy
ALDH7A1	Aldehyde Dehydrogenase 7A1
AR	Androgen Receptor
AR-V7	Androgen Receptor Variant 7
CoQ10	Coenzyme Q10
CRPC	Castration-Resistant Prostate Cancer
DAMP	Damage-Associated Molecular Pattern
FSP1	Ferroptosis Suppressor Protein 1
GPX4	Glutathione Peroxidase 4
GSH	Glutathione
GSSG	Glutathione Disulfide
LPCAT3	Lysophosphatidylcholine Acyltransferase 3
MBOAT1/2	Membrane-Bound O-Acyltransferase 1/2
MDA	Malondialdehyde
MSB	Menadione Sodium Bisulfite
MUFA	Monounsaturated Fatty Acid
NRF2	Nuclear Factor Erythroid 2-Related Factor 2
PCa	Prostate Cancer
PROTAC	Proteolysis-Targeting Chimera
PUFA	Polyunsaturated Fatty Acid
PUFA-PL	PUFA-containing Phospholipids
ROS	Reactive Oxygen Species
SFA	Saturated Fatty Acid
SLC7A11	Solute Carrier Family 7 Member 11
TME	Tumor Microenvironment
VPS34	Vacuolar Protein Sorting 34
